# Current Advances in Mechanisms and Treatment of Dry Eye Disease: Toward Anti-inflammatory and Immunomodulatory Therapy and Traditional Chinese Medicine

**DOI:** 10.3389/fmed.2021.815075

**Published:** 2022-01-17

**Authors:** Jiawei Ling, Ben Chung-Lap Chan, Miranda Sin-Man Tsang, Xun Gao, Ping Chung Leung, Christopher Wai-Kei Lam, Jiang-Miao Hu, Chun Kwok Wong

**Affiliations:** ^1^Institute of Chinese Medicine and State Key Laboratory of Research on Bioactivities and Clinical Applications of Medicinal Plants, The Chinese University of Hong Kong, Hong Kong, China; ^2^Department of Chemical Pathology, The Chinese University of Hong Kong, Prince of Wales Hospital, Hong Kong, China; ^3^Faculty of Medicine and State Key Laboratory of Quality Research in Chinese Medicines, Macau University of Science and Technology, Macau, Macau SAR, China; ^4^State Key Laboratory of Phytochemistry and Plant Resources in West China, Kunming Institute of Botany, Chinese Academy of Sciences, Kunming, China; ^5^Li Dak Sum Yip Yio Chin R & D Centre for Chinese Medicine, The Chinese University of Hong Kong, Hong Kong, China

**Keywords:** inflammation, signaling pathways, immunomodulatory therapy, traditional Chinese medicine, dry eye disease

## Abstract

Dry eye is currently one of the most common ocular surface disease. It can lead to ocular discomfort and even cause visual impairment, which greatly affects the work and quality of life of patients. With the increasing incidence of dry eye disease (DED) in recent years, the disease is receiving more and more attention, and has become one of the hot research fields in ophthalmology research. Recently, with the in-depth research on the etiology, pathogenesis and treatment of DED, it has been shown that defects in immune regulation is one of the main pathological mechanisms of DED. Since the non-specific and specific immune response of the ocular surface are jointly regulated, a variety of immune cells and inflammatory factors are involved in the development of DED. The conventional treatment of DED is the application of artificial tears for lubricating the ocular surface. However, for moderate-to-severe DED, treatment with anti-inflammatory drugs is necessary. In this review, the immunomodulatory mechanisms of DED and the latest research progress of its related treatments including Chinese medicine will be discussed.

## Introduction

The global prevalence of DED is 5 to 50%, and it increases linearly with age, with a higher prevalence among Asians (1–4). Common symptoms of DED include dryness of eye, foreign body sensation, burning sensation, itching, photophobia, eye redness, blurred vision, fluctuating vision and visual fatigue. In severe cases, corneal epithelial peeling, filiform adhesion and keratoconjunctival lesions can also occur. Traditionally, DED is defined as a disorder of the eye caused by tear film instability and ocular surface damage due to abnormal tear quality or quantity. In 2017, the TFOS DEWS II Working Group of the Tear Film and Ocular Surface Society (TFOS DEWS II) defined DED as a disorder of the ocular surface with a loss of tear film homeostasis and ocular symptoms caused by tear film instability, increased osmolarity, ocular surface inflammation, damage to the ocular surface and neurosensory abnormalities (5).

The ocular surface (cornea, conjunctiva and meibomian glands), the lacrimal gland, and the neural connections between them together form the Lacrimal Functional Unit (LFU), which regulates tear secretion, tear film formation and maintains the health of the ocular surface (6). Structural or functional damage to any component of the LFU can disrupt the integrity and function of the tear film, leading to dry eye disease (DED). Although the pathogenic mechanisms of DED has not been fully elucidated, ocular surface inflammation in DED becomes an important focus. Therefore, there have been relatively more studies investigating the roles of immune factors such as ocular surface inflammatory cells and inflammatory mediators in DED in recent years.

## Immunomodulation of Dry Eye Disease

### Immune Response

**Figure 1:**
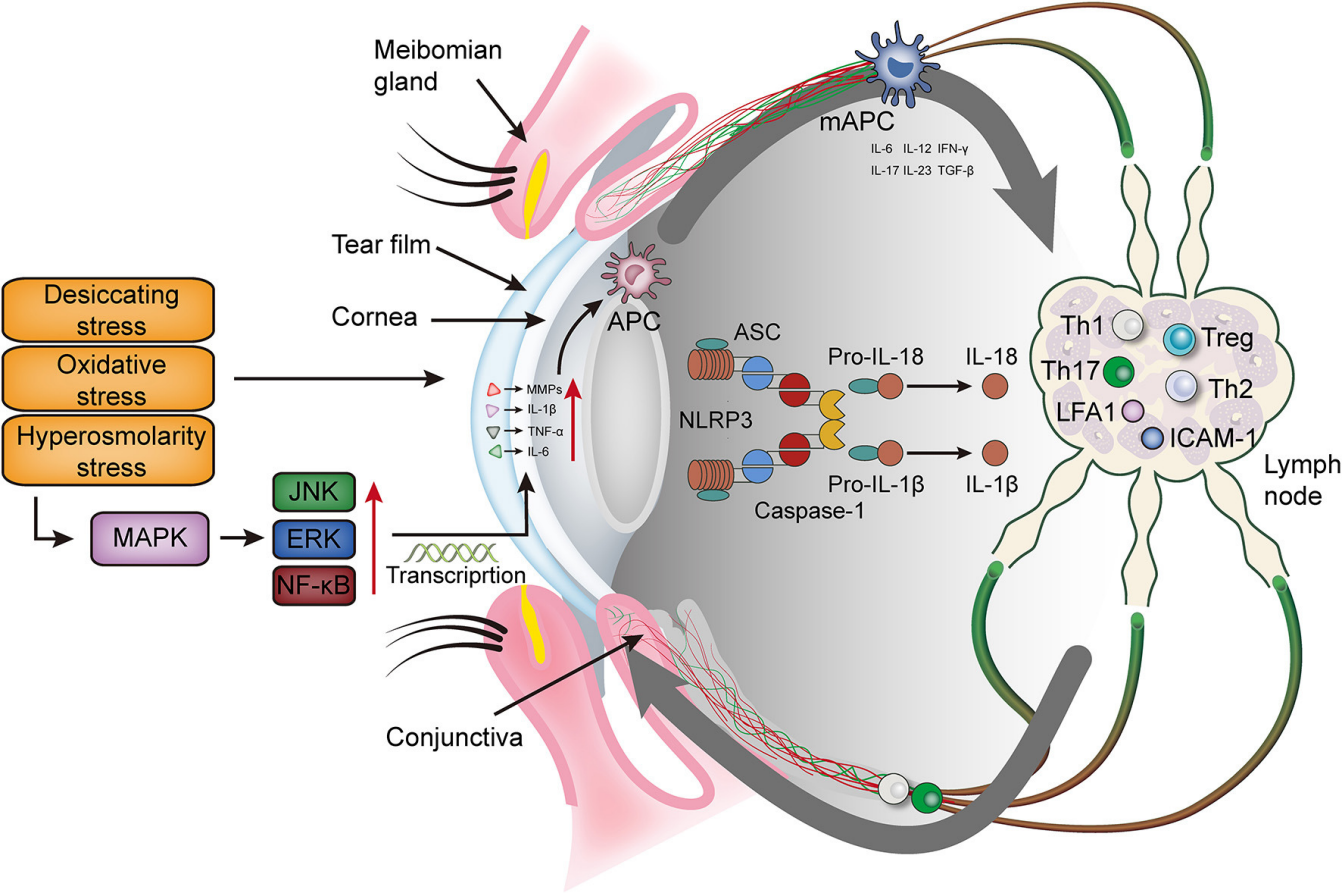
The immunoinflammatory response of the ocular surface in dry eye disease. Desiccating, oxidative and hyperosmolarity stress activate cell signaling pathways at the ocular surface, which leads to the production of pro-inflammatory cytokines (TNF-α, IL-1β and IL-6) and matrix metalloproteinase (mainly MMP9). These factors promote the maturation of antigen-presenting cells and allow mature antigen-presenting cells to migrate to the lymph nodes through the afferent lymphatic vessels. In the lymph nodes, mAPCs induce effector T cells (Th 1 and Th17) and recruit them to migrate to the ocular surface. Meanwhile, mAPCs activate the NLRP3 inflammasome, promotes the secretion of IL-1β and IL-18, and further aggravates the ocular surface inflammation. MAPK, mitogen-activated protein kinase; JNK, Jun N-terminal kinase; ERK, extracellular regulated protein kinase; NF-κB, nuclear transcription factor-κB; MMPs, matrix metalloproteinases; TNF-α, tumor necrosis factor-α; IL, interleukin; IFN-γ, interferon-γ; NLRP3, NLR family pyrin domain containing 3; TGF-β, transforming growth factor β; Th, T helper; Treg, regulatory T cell; LFA-1, lymphocyte function associated antigen 1; ICAM-1, intercellular adhesion molecule 1; APC, antigen presenting cell; mAPC, mature antigen presenting cell; ASC, apoptosis speck-like protein.

Both innate and adaptive immunity are tightly regulated in the ocular surface environment to protect and maintain ocular surface homeostasis (7). When this immune homoeostasis is being disrupted, it can lead to DED (8–12). Ocular surface immune responses, involving the participation of helper T (Th), memory T and regulatory T (Treg) lymphocytes, usually occur on the surface of the cornea, including the ocular tissues and regional lymph nodes (13). Innate immunity, also known as intrinsic immunity or nonspecific immunity, is gradually developed by organisms during long-term evolution. It is the body's front-line defense against pathogen invasion and can initiate and interact with adaptive immunity. Innate immune cells include monocytes, macrophages, neutrophils, dendritic cells, natural killer cells (NK cells), γδ T lymphocytes, etc. (14–16). Adaptive immunity, also known as acquired or specific immunity, is highly specific to a particular pathogenic microorganism (17). Innate immunity and adaptive immunity are complementary and inseparable (Table 1) (34). Unlike other mucosal surfaces, the ocular surface microenvironment is continuously exposed to the environmental factors and helps to monitor the invasion of microorganisms, contaminants, allergens and other harmful substances. The immune system respond accordingly to the acute or chronic nature of the invasion process of the ocular surface in DED (Figure 1).

**Table 1:** Cell populations present in innate and adaptive immune responses in dry eye disease.

**Immune response**	**Cell type**	**Related indicators that DED individuals differ from healthy individuals**	**Reference**
Innate response	neutrophils	IL-1β**↑** IL-6**↑** MMPs**↑** CXCL1**↑**	(18)
	macrophages	CD68^+^ macrophages**↑** IL-18**↑** iNOS**↑**	(19–21)
	NK Cells	IL-4**↑** IL-5**↑** IL-13**↑** IFN-γ**↑**	(22–24)
	eosinophils	Infiltration (not exist in normal eyes)**↑** Th2 inflammation**↑**	(25)
Adaptive response	CD4^+^ T cells	Intercellular adhesion molecule 1 (ICAM-1)**↑** CXCL11**↑** CD4^+^ T cells infiltration**↑**	(26, 27)
	CD8^+^ T cells	ICAM-1**↑** CD8^+^ T cells↑	(28)
	Th17	IL-6**↑** IL-23R**↑** IL-21**↑** IL-22**↑** TGFβ2**↑** IL-17**↑** CCR6**↑** CXCR3**↑**	(29, 30)
	B cells	Cell population and numbers—IL-17**↑**	(31–33)

### Innate Immune Response of the Ocular Surface

#### Barrier and Inflammatory Signals

A key function of the innate immune system is to provide a physical barrier between the eye and the external environment, and thus to prevent the microbial attachment and toxins from crossing the surface epithelium. Components responsible for this function include mucins in the tear fluid, glycocalyx, corneal and conjunctival epithelium, and a series of anti-microbial defense proteins (lactoferrin, lysozyme, lipid transport proteins, tripeptides, defensins) (35).

Existing studies have shown that a large number of lymphocytes infiltration can be seen in the lacrimal gland and conjunctival tissue of dry eye patients (36). The infiltrated inflammatory cells produce pro-inflammatory cytokines. The secretion of natural anti-inflammatory factors (lactoferrin) in the tear fluid is reduced, and the scope of inflammation is gradually expanded (37, 38). At present, the pathogenic mechanism of dry eye has not been fully elucidated, but inflammation is closely related to the occurrence and development of the dry eye. Inflammation can affect the stability of the tear film on the ocular surface, thereby increasing the tear osmotic pressure (39). These changes further induce damage to the ocular surface and initiate an inflammatory cascade of innate and adaptive immune responses.

In 2007, the international dry eye workshop (DEWS) introduced the increase in tear osmotic pressure into the concept of dry eye, indicating that the increase in tear osmotic pressure is an important feature of dry eye pathological damage. Tomlinson et al. (40) used an osmotic pressure exceeding 316 mOsm/L as the diagnostic criteria for dry eye, and found that the sensitivity and specificity were 59 and 94% respectively. Suzuki et al. (41) evaluated the symptoms and signs of 19 dry eye patients and found that the increase in tear osmotic pressure was highly correlated with the severity of dry eye. Increased tear osmotic pressure in patients with dry eye can induce ocular surface inflammation. VanDerMeid et al. (42) found that increased tear osmotic pressure is positively correlated with cytokines (IL-1α, IL-6, TNF-α) and matrix metalloproteinase 2, 9 and 10. The hyperosmotic state of DED can disrupt this defense system by activating various signaling pathways; for instance, mitogen-activated protein kinase (MAPK), c-Jun N-terminal kinases (JNKs), extracellular regulated protein kinases (ERKs) pathways and in particular, the p38 pathway, which in turn activates nuclear transcription factor-κB (NF-κB), leading to the release of interleukin (IL)-1 and tumor necrosis factor-α (TNF-α). This inflammatory mechanism can then induce the release of other downstream mediators and the activation of cellular signaling that amplify inflammatory response (43, 44). In addition, the activation of pathogen-related pattern recognition receptors (PRR) is also involved in the inflammatory response of DED (45). In innate immune cells, Toll-like receptors (TLRs)-3,7 & 9 and RNA sensors-mediated signaling can lead to the activation of inflammatory caspases within signaling platforms called inflammasomes, with subsequent maturation and secretion of a variety of pro-inflammatory cytokines such as IL-1β and IL-18 [38].

#### Inflammatory Mediators

The expression of inflammatory IL-1β, TNF-α and IL-6 of the ocular surface epithelium is critical for the inflammatory response in DED. Knockdown of IL-1 receptors in mice results in a significant reduction in inflammatory factors produced by the cornea and conjunctiva (46). In the ocular surface, chemokines such as CC chemokine ligand (CCL)3, CCL4, CCL5, CXCL9, C-X-C motif chemokine ligand (CXCL)10 and CX3CL1 can recruit macrophages, dendritic cells, neutrophils, activate T cells and upregulate the corresponding chemokine receptors (47, 48). Another key factor is the expression of intercellular adhesion molecule 1 (ICAM-1) in corneal and conjunctival epithelium, which binds to its ligand lymphocyte function associated antigen 1 (LFA-1) at sites of inflammation and lymph (49). Innate and adaptive immune responses occur in different regions of the ocular surface, but they share common key molecular interactions that promote cell migration. The interaction between ICAM-1 and LFA-1 is critical for immune cell proliferation and infiltration, and can be a potential therapeutic target.

The cells involved in the innate immune response in DED are mainly neutrophils, NK cells and monocytes/macrophages. Neutrophils constitute the first line of defense for the host innate immune response. Although they are low in number in the normal conjunctiva, they are abundant on the ocular surface of patients with severe aqueous-deficient dry eye (50). Neutrophil depletion leads to the increased CD4+ Th cell activation and the increased extent of corneal staining, suggesting that neutrophils may play certain protective role (51). A recent study has suggested that NK cells may play an important role in the pathogenesis of DED (22). The recruitment or activation of NK cells at the ocular surface enhances the production of inflammatory cytokines such as interferon-γ (IFN-γ), IL-6 and Th17-related IL-17, which stimulate macrophages, antigen presenting cells (APC) and autoreactive T lymphocytes. IFN-γ is responsible for T helper 1 (Th1) cell activation and differentiation, and can cause cell-mediated conjunctival epithelial damage and goblet cell loss (26). Monocytes infiltrated to the conjunctiva can be differentiated into two subtypes of macrophages: M1 cells associated with pro-inflammatory responses and M2 cells responsible for immunoregulation. Studies have shown that DED mainly induces the differentiation of monocytes toward the M1 phenotype (52). The innate immune system also includes γδ T cells and the complement system. The γδ T cells are often present near epithelial cells, and they can produce IL-17 at the ocular surface, but their specific role for DED remains unknown. Studies on the role of complement in ocular surface inflammation in DED have been limited to the observations in animal models, where nude mice receiving serum from mice with DED can develop DED with the recruitment of inflammatory cells and production of cytokines induced by complement C3a/C5a and C3b/C5b activation (53). In addition to the above factors, inflammatory mediators associated with DED include IL-2, IL-4, IL-5, CXCL8, matrix metalloproteinase 3, macrophage inflammatory protein 2, epidermal growth factor, lactoferrin, transforming growth factor (TGF), mucin MUC5AC and S100 protein. Changes in these inflammatory factors can be detected in the ocular surface of DED patients. Most of the above inflammatory factors are found in dry syndrome type of DED and the changes are more pronounced on the ocular surface (54).

### Adaptive Immune Response of the Ocular Surface

The presence of CD4+ Th cells on the ocular surface in DED and the efficacy of topical cyclosporine in treating ocular surface inflammation provide valid evidence for the involvement of the adaptive immune response in DED. Adaptive immunity mainly involves the production and recruitment of effector T cells (55, 56). The initiation of an adaptive immune response requires antigens at the site of inflammation to be processed and presented by APC, which then migrate to the lymphoid tissue to activate and induce the proliferation of specific effector T cells (57). Although the antigen that initiates this response remains unknown, study has suggested that the expression of autoantigens is critical for triggering the inflammatory epithelial lesions in DED (58). Since the ocular surface in inflammatory states is characterized by the upregulation of major histocompatibility complex class II (MHCII) and other stimulatory signals (IL-6 and IL-17 etc.), the recruitment of activated T cells to the conjunctiva and cornea of patients with DED may be an alternative pathway for antigen presentation in the adaptive immune response (53). After mature APC and CD4+ T cells combine in the lymph nodes through immune synapses (59), they secrete IL-6, IL-12, IL-17, IL-23, TGF-β and IFN-γ, together with pro-inflammatory cytokines from the corneal epithelial cells, promote the differentiation of CD4+ T cells into Th1, T helper 2 (Th2), T helper 17 (Th17) and Treg cells (60). Effector T cells then undergo migration and recruitment to the conjunctival stroma (61) for the activation of residual APC, in turn induce the damage to the ocular surface epithelium and dysfunction through the release of inflammatory cytokines IL-1β, TNF-α and IL-6 (62–65). Th1 and Th17 are the main Th lymphocyte subsets that account for ocular surface dysfunction (66, 67). IFN-γ secreted by Th1 cells promotes the apoptosis of ocular surface epithelial cells and the loss of conjunctival goblet cells (68, 69). IL-17 secreted by Th17 cells promotes corneal epithelial cells and fibroblasts to produce matrix metalloproteinases (MMPs), which leads to the destruction of the corneal epithelial barrier and further damage of the ocular surface (70–72).

Although the spleen is the primary lymphoid tissue responsible for intraocular immune regulation, its role in ocular surface inflammation is not predominant. The role of the thymus in the regulation of ocular surface immune responses is unknown. However, animal models of DED and the observation of the ocular surface status of patients with graft-vs.-host disease causing thymic damage prior to hematopoietic stem cell transplantation suggest that the central tolerance regulated by the thymic environment is important for ocular surface immunity (73). Conjunctival-associated lymphoid tissue that reaches the epithelial cell surface will respond according to local antigen exposure to form germinal centers, which can take part in mucosal tolerance as well as immune defense against ocular surface inflammation (74).

### Pyroptosis

Pyroptosis, also known as cellular inflammatory necrosis, is a newly discovered form of cell death that is important to innate immune response. It is manifested by the continuous expansion of cells until the cell membrane ruptures, leading to the release of cellular contents and thus the activation of an intense inflammatory response (75). Cell death is dependent on caspase-1 and its PRR, NLR family pyrin domain containing 3 (NLRP3) inflammasome, which cleaves the inactive caspase-1 precursor to be active caspase-1 (76). Caspase-1 can induce cell membrane perforation to cause cell lysis and death, with the subsequent release of intracellular materials through the disrupted cell membrane to induce an inflammatory response. Caspase-1 can also cleave the IL-1β and IL-18 precursors to produce and secrete the active mature IL-1β and IL-18. IL-18 then induce the synthesis and release of other inflammatory cytokines and amplify local and systemic inflammatory responses (77). It has been demonstrated that the expression of mRNA and protein of NLRP3, caspase-1, IL-1β and IL-18 is elevated in the ocular surface of patients with DED (78).

### The Meibomian Gland and the Immune Response

A distinguishing feature of the human meibomian gland is its resistance to inflammation and infection. When keratoconjunctival epithelial cells are exposed to bacterial toxins, they cause the significant upregulation of immune-related genes, cytokine and chemokine expression, TLR signaling pathways, and inflammatory and immune responses. However, epithelial cells of the meibomian gland do not upregulate pro-inflammatory gene expression and TLR signaling upon the exposure to bacterial toxins (79), leading to the speculation that the meibomian gland may have inherent anti-inflammatory and anti-infective factors. The most highly expressed gene in the meibomian gland is the leukocyte-associated immunoglobulin-like receptor, an inhibitory receptor that inhibits immune cell activation and reduces the production of pro-inflammatory cytokines (80). Moreover, the immunoglobulin-like receptor gene expression is upregulated upon epithelial cell differentiation in the human meibomian gland (81). Recently, researchers have also found that human meibomian gland epithelial cell lysates inhibit the growth rate of gram-negative bacteria and pseudomonas aeruginosa (57).

### Immunoinflammatory Pathways Induced by Desiccating Stress

Ocular surface desiccating stress can induce autoreactive T cells. When the patient has immunodeficiency, it can cause Sjogren's syndrome-like inflammation in the lacrimal gland, cornea and conjunctiva, but it does not interfere with other organs (82). The desiccating stress acting on the ocular surface activates intracellular signal transduction pathways as a trigger condition to release pro-inflammatory factors that promote the maturation and activation of immature APC. The infiltration of APC to lymph nodes through lymphatic vessels, together with the subsequent migration of Th1 and Th17 cells to the ocular surface. IFN-γ and IL-17 together exert pathogenic properties and cause squamous metaplasia of ocular surface epithelial cells. IFN-γ decreases goblet cell differentiation and IL-17 disrupts corneal epithelial barrier function, while IFN-γ and IL-17 also enhance the production of pro-inflammatory cytokines, chemokines, MMPs, cell adhesion molecules and vascular endothelial growth factor. These inflammatory mediators again promote the maturation and migration of APC. This creates a vicious cycle of inflammation that ultimately leads to the destabilization of the ocular surface and tear film, as well as an increase in tear osmolarity (83, 84).

## Anti-Inflammatory Treatment for Dry Eye Disease

The complications of dryness in DED may vary from a serious abnormal feeling sensation to contact lens intolerance (85). Currently, the methods of treating DED worldwide mainly include the application of ocular surface lubricants to protect the mucous membrane and the use of anti-ocular surface inflammation drugs, punctual plug placement to reduce tear loss, and physical therapy of the eyelids to restore the meibomian glands (86). However, various methods can only reduce but not completely eliminate the symptoms of DED. Artificial tears (87), silicone eye shields (88), gas lenses (89) and corneal contact lenses (90) are the basic treatment for DED, but in moderate-to-severe DED with ocular surface inflammation, anti-inflammatory and immunosuppressive treatments are essential (Table 2) (128).

**Table 2:** Anti-inflammatory and immune-related therapeutics associated with dry eye disease.

**Group**	**Drug name**	**Clinic/Trail dosage**	**Administration method**	**Properties**	**Reference**
Antibiotics	Azithromycin	1%	Topical administration	good tolerance, alleviate symptoms of dry eye and restore tear film	(91, 92)
	Tetracycline	0.05%	Topical administration	inhibit MMPs, downregulate inflammatory cytokines, and promote the recovery of meibomian gland function	(93)
	Doxycycline	0.03%	Topical administration	downregulate IL-1β, IL-6 and MMPs significantly, alleviate symptoms of dry eye, improve meibomian gland function	(18, 94, 95)
		100 mg	Oral administration		
	Minocycline	50 mg, 100 mg	Oral administration	inhibit the growth of bacteria, improve the MGD, but reduce the secretion of tear volume	(94, 96)
Non-steroidal anti-inflammatory drugs (NSAIDs)	Ketorolac tromethamine	0.40%	Topical administration	downregulate inflammation, suitable for ocular infections caused by allergic conjunctivitis and inflammation after various ophthalmic operations	(97, 98)
	Pranoprofen	0.10%	Topical administration	relieve dry eye symptoms, reduce inflammatory factors, good tolerance	(99, 100)
	Diclofenac	0.10%	Topical administration	improve dry eye symptoms, reduce inflammation, good absorption	(101, 102)
	Phospho-sulindac (OXT-328)	0.05%-1.6%	Topical administration	suppress NF-κB pathway, inhibit IL-6, CXCL8 and MMPs activity	(103, 104)
	Flurbiprofen	0.03%	Topical administration	reduce inflammation, inhibit the expression of IFN-γ and TNF-α, and relieve dry eye symptoms	(105)
	Nepafenac	0.10%	Topical administration	inhibits cyclooxygenase 2, has no obvious effect on the production of inflammatory factors and tears, and is not suitable for severe dry eye	(106, 107)
	Bromfenac	0.10%	Topical administration	reduce inflammation, good tolerance, will not affect eye sensitivity and tear secretion	(108, 109)
Glucocorticoids	Methylprednisolone	0.10%	Topical administration	improve dry eye symptoms, reduce tear osmotic pressure, and inhibit the expression of pro-inflammatory cytokines	(110, 111)
	Dexamethasone	0.10%	Topical administration	downregulate IL-1β, IL-6 and MMPs significantly, alleviate the symptoms of dry eye, stabilize tear film and improve MGD	(18, 112)
	Fluorometholone	0.10%	Topical administration	activate glucocorticoid receptors, relieve dry eye symptoms, reduce the deterioration caused by desiccating stress and enhance the expression of mucin	(113–115)
	Loteprednol	0.25%	Topical administration	relieve dry eye symptoms, good tolerance, stabilize tear film	(116, 117)
Immuno-suppressants	Cyclosporine A	0.05%	Topical administration	inhibit the expression of pro-inflammatory cytokines, improve anti-inflammatory activity, alleviate dry eye symptoms and stabilize tear film	(118, 119)
	Tacrolimus	0.01%, 0.03%	Topical administration	improve ocular surface condition and tear secretion, relieve dry eye symptoms, suitable for dry eyes caused by Sjogren's syndrome	(120, 121)
	Voclosporin	0.20%	Topical administration	inhibit the expression of pro-inflammatory cytokines, relieve the symptoms of dry eye and reduce the loss of goblet cells	(122, 123)
LFA-1 antagonists	Lifitegrast	5%	Topical administration	inhibit the activation and proliferation of T lymphocytes, reduce the release of inflammatory mediators and relieve the symptoms of dry eye	(124, 125)
Thymosin β4	RGN-259	0.10%	Topical administration	significantly alleviate the symptoms of dry eye, with a larger safety window, with no side effects	(126, 127)

### Tetracycline and Its Derivatives

Tetracycline and its derivatives are broad-spectrum antibiotics with anti-inflammatory properties. Tetracycline can reduce the activity of collagenase and phospholipase A2, inhibit the activity of MMP-9 in the ocular surface tissues, and downregulate the expression of inflammatory cytokine IL-1β and TNF-α. Doxycycline and minocycline are commonly used ocular surface anti-inflammatory drugs, they are both tetracycline derivatives. In an experimental mouse model of DED, topical application of doxycycline has soothing effects, function as a barrier to the corneal epithelium and reduces ocular surface inflammation (129). Tetracycline and its derivatives are commonly used in the treatment of DED caused by meibomian gland dysfunction (MGD) and blepharitis. There is no consensus standard dosage at present, but doctors can make adjustments on the dosage according to the patient's condition.

### Non-steroidal Anti-inflammatory Drugs (NSAIDs)

Non-steroidal anti-inflammatory drugs overcome the defects of many adverse reactions and strong drug dependency of corticosteroid drugs. NSAIDs such as ketorolac tromethamine can inhibit the activity of cyclooxygenase, block the synthesis of prostaglandin, reduce the migration and phagocytosis of granulocytes and monocytes, and achieve the purpose of controlling ocular surface inflammation. However, for DED caused by autoimmune diseases such as Sjogren's syndrome, the immunomodulatory activity of NSAIDs is much lower than that of glucocorticoids (130). Moreover, some NSAIDs have reduced corneal sensitivity in both normal subjects and patients with DED (131). Therefore, NSAIDs are not recommended for patients with mild DED dominated by environmental factors (low humidity and smoke exposure, etc.) (132).

Pranoprofen is a propionic acid-derived NSAID that can relieve the symptoms of DED. It is currently used widely in ophthalmology clinics. Liu et al. (99) found that the combination of pranoprofen and sodium hyaluronate can quickly control the symptoms of DED, effectively stimulate the secretion of tears, and achieve significant clinical effects. It may be related to the fact that pranoprofen reduces the level of inflammatory mediators and prevents the accumulation of leukocytes in eyes. Diclofenac sodium is a typical non-steroidal anti-inflammatory drug, which has antipyretic, analgesic and anti-inflammatory effects to improve vision, reduce photophobia and alleviate the clinical symptoms of DED. It can be completely absorbed, with full infiltration into the eye tissue after 30 min of medication (133). Studies have shown that diclofenac eye drops combined with sodium hyaluronate in the treatment of DED can improve eye symptoms significantly, with good safety without irritation and induction of infection upon long-term use (101). Bromfenac sodium eye drops have good penetrability, the abilities to maintain the blood concentration of the ocular surface for a long time and improve the inflammatory response of patients with DED (108). It has been shown that while bromfenac sodium eye drops not only relieve the symptoms of DED but also promote the improvement of the patient's lacrimal gland function and prevent the development of inflammation (134).

### Glucocorticoids

Glucocorticoids have a rapid onset of action, including the inhibition of the inflammatory factor production, downregulation of pro-inflammatory mediator IL-1 and TNF-α by inhibiting the NF-kB signal pathway, and induction of lymphocyte apoptosis. Methylprednisolone is a synthetic corticosteroid and Methylprednisolone (1%) can inhibit the expression of MMP-9 and MAPK activity in the corneal epithelium of mice with DED. The use of glucocorticoids for 2–4 weeks can significantly improve the symptoms of DED, but its long-term use can cause complications such as high intraocular pressure and cataracts (135).

Flumetholone is a glucocorticoid drug with a high rate of clinical use. Flumetholone can effectively neutralize inflammatory mediators, improve non-infectious inflammatory symptoms, and decrease lymphocytes and macrophages activation, thereby enhancing the anti-inflammatory effect. Fluorometholone eye drops (0.1%) is safe and has low incidence of adverse effects (136). Loteprednol is a new type of glucocorticoid drug (137) that has recently been approved by the FDA for the treatment of short-term DED (138). Loteprednol can fully exert therapeutic effects in DED by inducing the inhibition of phospholipase A. Loteprednol has low toxicity with high fat-solubility and good corneal permeability.

Korenfeld et al. (139) found that loteprednol has a significant clinical effect in the treatment of moderate-to-severe DED. For examples, it can effectively improve dry eye symptoms, promote the recovery of corneal damage and stabilize the tear film of DED patients.

### Immunosuppressants

Cyclosporine A (CsA), an immunosuppressant, is a fungal metabolite that inhibits Th1-related IL-2 activation. It has been used in anti-rejection therapy for organ transplantation, autoimmune diseases, local allergy, corneal limbal stem cell dysfunction and autoimmune uveitis. CsA (0.05%) was approved by the FDA in 2003 for the treatment of moderate-to-severe DED (140), as it can reduce inflammatory cells and inflammatory mediators on the ocular surface, inhibit the apoptosis of lacrimal cells and conjunctival goblet cells, promote lymphocyte apoptosis, and reduce the ocular surface inflammatory response. It has been shown that CsA eye drops combined with vitamin A can significantly increase the amount of tear secretion in dry eye rats, prolong the tear film rupture time, and alleviate the apoptosis of corneal cells (141). Tacrolimus is a new type of immunosuppressant with the same action as CsA, but with stronger anti-inflammatory effects and fewer side effects than CsA. It has been shown that tacrolimus can effectively alleviate the pathological changes of conjunctival epithelial cell hyperplasia and goblet cell reduction caused by Sjogren's syndrome in mice (142). Moscovici et al. (120) used tacrolimus (0.03%) to treat patients with Sjogren's syndrome, suggesting that tacrolimus in the form of eye drops may have positive effects when administered locally for treating severe autoimmune DED caused by Sjogren's syndrome. It has also been shown that tacrolimus is effective in treating dry eyes caused by Sjogren's syndrome, Stevens-Johnson syndrome, graft-vs.-host disease and diabetes (140, 143).

### LFA-1 Antagonists

The main ligand of LFA-1 is ICAM-1, which is expressed on the cell surface of endothelial cells, epithelial cells and APC (144). The binding of LFA-1 to ICAM-1 is known as the “immune synapse” and is the key step in T-cell activation. Lifitegrast is a new type of small molecule T lymphocyte inhibitor, which can effectively suppress the migration of APC, as well as the migration and recruitment of T lymphocytes to the ocular surface by inhibiting the interaction between LFA-1 and ICAM-1 (145). Lifitegrast is structurally similar to ICAM-1 and acts as a competitive antagonist to block LFA-1 and ICAM-1 binding and hence reduce the release of inflammatory factors (145). Lifitegrast (5%) reduces dry eye scores and ocular discomfort with a good safety and tolerability profile (146). Experimental studies have shown that Lifitegrast can inhibit the levels of IL-1β, IFN-γ and IL-10, and can increase the number and spread of conjunctival goblet cells (147). It was approved by the FDA in 2016 to be a novel drug for the treatment of DED (146).

### Thymosin β4

RGN-259 is a natural polypeptide composed of 43 amino acids, which is the main protein of wound repairing cells (platelets, macrophages and polymorphonuclear cells) (148). For the treatment of DED and neurotrophic keratopathy, RGN-259 is a new preservative-free peptide eye drops with Thymosin β4 (Tβ4) as the active ingredient developed by RegeneRx. Preclinical studies have shown that Tβ4 can promote the migration of corneal epithelial cells, increase cell-interstitial contact, reduce cell apoptosis and reduce corneal inflammation. The Phase II clinical trial (ARISE-2) ended in October 2017 showed that RGN-259 (0.1%) provides ocular comfort, is safe and highly tolerable (149). The symptoms of DED were significantly improved 15 days after treatment, and corneal staining was also significantly improved 15 days and 29 days after treatment (150).

### Biological Agents

In recent years, several biological agents have also been shown to reduce ocular surface inflammation, improve dry eye symptoms and corneal staining. These biologicals include lubricating agents, recombinant human nerve growth factor, IL-1 receptor antagonists, anti-TNF-α agents, anti-IL-17 agents, calcitonin gene-related peptides and neuropeptide Y (140). Although many biologic agents have been thoroughly studied in animal models, clinical studies in humans are still scarce and human clinical trials with randomized, double-blind and placebo-controlled Good Clinical Practice are needed to further evaluate their therapeutic effects.

### Traditional Chinese Medicine (TCM)

China has a long history of using traditional Chinese medicine to treat DED. Long-term clinical practice has confirmed that TCM has a pronounced systemic therapeutic effect on DED. In recent years, in-depth research has also proven the multi-targeted efficacy of TCM extracts in treating a variety of DED endotypes. The reported TCM for the treatment of the dry eye disease is summarized in the Table 3. Zhang *et al*. investigated the effect of extracts of Bidens pilosa L. on tear secretion in dry eye rats induced by finasteride, they found that Bidens pilosa L. extracts could increase tear production and prolong breakup time (BUT). Moreover, Bidens pilosa L. extracts can increase the tear film stability, inhibit the inflammatory response of the lacrimal gland and reduce the pro-inflammatory cytokines (151). A series of animal experiments confirmed that the extract of Buddleja offcinalis is effective for the treatment of rabbit dry eye model, and the extract of Buddleja offcinalis has the effect of inhibiting the expression of inflammatory cytokines TNF-α and IL-1β. The extract of Buddleja offcinalis can significantly improve the pathological changes of the lacrimal gland tissue, increase the production of tears and the stability of the tear film. The extract of Buddleja offcinalis can also inhibit the apoptosis of lacrimal gland cells and inhibit the expression of IL-1β and TNF-α in the lacrimal gland tissue (152). The main component of Buddleja offcinalis is flavonoids, which have a similar effect to androgens, so its extracts can improve the ocular surface symptoms in castrated male rats with dry eyes (153). Yao XL *et al*. studied the efficacy of total flavonoids from chrysanthemum on castration-induced dry eye in male rabbits and its effect on the expression of Bax and Bcl-2 in lacrimal gland cells and found that total flavonoids from chrysanthemum can significantly inhibit the occurrence of the dry eye accompanied with the apoptosis of lacrimal gland cells and maintain the stability of the tear film (154). The total flavonoids of chrysanthemum have an effect on the pro-apoptotic factor Bax in lacrimal gland cells and the activation of the anti-apoptotic factor Bcl-2, thereby inhibiting the apoptosis of lacrimal epithelial cells and improving ocular surface symptoms (155). In a rat model of dry eye induced by particulate matter 2.5 (PM2.5), Lee *et al*. found that the water extract of Corni Fructus can alleviate the decrease in tear secretion and corneal epithelial damage. In addition, the water extract of Corni Fructus can inhibit the apoptosis of conjunctival goblet cells and down-regulate the expression of inflammatory factors in the lacrimal gland. Corni Fructus water extracts help to improve retinal function and lipid metabolism disorders (156). Park et al. (158) used polydatin eye drops to treat dry eye models in rats with lacrimal gland excision and found that polydatin eye drops can improve tear reduction and repair corneal damage. Polydatin eye drops can reduce tear film BUT and reduce goblet cell loss. In addition, polydatin can reduce the expression of inflammatory cytokines and NLRP3 in conjunctival tissues (159). According to Chinese medicine, middle-aged and elderly people are prone to the symptoms of liver and kidney “Yin” deficiency, which are manifested as dryness in the eyes and foreign body sensation. There are many descriptions for middle-aged and elderly dry eye syndrome, especially in menopausal women. Prunella vulgaris exerts the effect of lowering blood pressure and cleaning the liver. It has been shown that the administration of Prunella vulgaris can significantly improve the diagnostic indicators of dry eye in menopausal women. Prunella vulgaris can reduce the cytokine production of TNF-α and IL-1β, and reduce ocular surface inflammation (161). Lycium barbarum belongs to the genus Lycium barbarum of the Solanaceae family which is the mature fruit of its plant. Chinese medicine believes that Lycium barbarum has the effects of nourishing “Yin”, repairing the liver and improving eyesight. Lycium barbarum is rich in vitamin A and carotene, which are the components of rhodopsin in the visual cells that sense slight light, and play an important role in maintaining normal visual function. Liu *et al*. found that in human retinal epithelial cells and dry eye rat models, Lycium barbarum can not only improve the stability of the tear film, promote tear secretion, but also repair the cornea, reduce oxidative stress, and improve the secretion function of the meibomian glands (168, 169).

**Table 3:** Traditional Chinese medicines used for the treatment of the dry eye disease.

**Scientific name**	**Family**	**Part used**	**Form of extract**	**Properties**	**Reference**
*Bidens pilosa* L.	Asteraceae	leaves	water extract	Alleviate the symptoms of dry eye (the BUT and tear quantity), downregulate the pro-inflammatory cytokines IL-1β and TNF-α	(151)
*Buddleja officinalis* Maxim.	Scrophulariaceae	flower buds	ethanol extract	Inhibit the apoptosis of lacrimal gland cells, promote the secretion of tears and maintain the stability of the tear film, downregulate apoptotic factors Bax and Fas	(152, 153)
*Chrysanthemum × morifolium* Ramat	Asteraceae	flower buds	total flavonoids	Inhibit the apoptosis of lacrimal gland cells, promote the secretion of tears and maintain the stability of the tear film, downregulate apoptotic factors Bax and Fas	(154, 155)
*Cornus officinalis* Sieb. et Zucc.	Cornaceae	fruits	water extract	Alleviate the symptoms of dry eye, increase tear secretion and promote anti-inflammatory effect, inhibit expression of IL-1β, IL-6 and TNF-α in conjunctiva and cornea	(156)
*Houttuynia cordata* Thunb.	Saururaceae	cauline leaves	water extract	Improve the symptoms of dry eye (the BUT and tear quantity), suitable for mild-to-moderate MGD-related DED	(157)
*Polygonum cuspidatum* Sieb.et Zucc.	Polygonaceae	root and rhizome	polydatin, water extract	Increase tear secretion, repair corneal damage, improve BUT, reduce goblet cell loss, inhibit the expression of pro-inflammatory cytokines and NLRP3	(158–160)
*Prunella vulgaris* L.	Lamiaceae	fruit-spike	water extract	Inhibit IL-1β, TNF-α and ICAM-1 in conjunctival epithelial cells, suitable for menopausal women with dry eye syndrome	(161)
*Hippophae rhamnoides* L.	Elaeagnaceae	fruits and seeds	oil extract	Maintain the tear film osmolarity, promote tear secretion, inhibit inflammatory cytokines in the lacrimal gland	(162–164)
*Prunus armeniaca* L.	Rosaceae	seeds	water extract	Promote tear secretion, stabilize the ocular surface, increase the expression of mucin, suppress pro-inflammatory cytokines IL-6 and TNF-α	(165, 166)
*Rhynchosia volubilis* Lour.	Fabaceae	seeds	ethanol extract	Improve the symptoms of dry eye (the BUT), downregulate Bax	(167)
*Lycium barbarum* L.	Solanaceae	fruits	water extract	Alleviate the symptoms of dry eye (the BUT and tear quantity), inhibit oxidative stress and inflammation	(168–170)
*Dendrobium officinale* Kimura et Migo	Orchidaceae	stems	water extract, polysaccharides	Alleviate the symptoms of dry eye in rats and inhibit the expression of IL-1β and TNF-α, upregulate AQP5 and MUC5AC in conjunctival cells	(171–173)

Based on our previous research findings, the water extract of *Dendrobium officinale* can relieve the symptoms of DED in rats and inhibit the expression of IL-1β and TNF-α (171, 172). Aquaporins (AQPs) participate in the water transport across cell membranes driven by osmotic pressure, so as to maintain the balance of the volume of body cavity fluid and saline. Various studies have shown that the expression of AQP5 in ocular tissues was reduced in the state of dry eye. Our results demonstrated that the water extract of *Dendrobium officinale* possess therapeutic effects in treating DED via activating and upregulating AQP5 and MUC5AC in conjunctival cells (172). Dry eye disease not only induces symptoms on the ocular surface but also systemic symptoms. With the continuous in-depth study of dry eye syndrome in traditional Chinese medicine, besides monomeric Chinese herbal extracts, Chinese herbal formulas and decoctions are also commonly used methods. Therefore, when modern medicine has not achieved a particularly ideal treatment for dry eye disease, we should give full play to the advantages of traditional Chinese medicine to assist modern medicine in the joint treatment of dry eye disease. The combination of traditional Chinese medicine and Western medicine can often achieve better results.

## Conclusion

The pathogenesis of DED is complex and multifactorial. Currently, inflammation at the ocular surface is the main cause of DED. The corresponding anti-inflammatory pathways are the key targets in DED treatment. On the other hand, current animal models and *in vitro* studies have confirmed that inflammatory cells and inflammatory factors are closely associated with the tear film instability and the elevated tear osmolarity. Differential activation of distinct intracellular signal transduction pathways will lead to the release of inflammatory factors from inflammatory immune cells. These inflammatory mediators will then promote the infiltration of more pro-inflammatory cells to the local inflammatory sites, thus forming a vicious cycle of inflammation. Although a more detailed mechanisms of ocular surface inflammation requires further investigation, blocking the vicious cycle of ocular inflammation by suppressing various signaling pathways, or the application of multi-targeted TCM could be the tactics in the treatment of DED.

## Author Contributions

JL and BC reviewed the literature and wrote the manuscript. MT, XG, PL, CL, and J-MH reviewed and revised the manuscript. CW reviewed the literature and contributed to the conceptualization of the manuscript. All authors contributed to the article and approved the submitted version.

## Funding

This study was supported by State Key Laboratory of Research on Bioactivities and Clinical Applications of Medicinal Plants, CUHK from Innovation and Technology Commission, Hong Kong and funding from Li Dak Sum Yip Yio Chin R&D Center for Chinese Medicine, the Chinese University of Hong Kong.

## Conflict of Interest

The authors declare that the research was conducted in the absence of any commercial or financial relationships that could be construed as a potential conflict of interest.

## Publisher's Note

All claims expressed in this article are solely those of the authors and do not necessarily represent those of their affiliated organizations, or those of the publisher, the editors and the reviewers. Any product that may be evaluated in this article, or claim that may be made by its manufacturer, is not guaranteed or endorsed by the publisher.

## Abbreviations

APC/mAPC: antigen presenting cell/mature antigen presenting cell
AQP: aquaporin
BUT: break-up time
CsA: cyclosporine A
CCL: CC chemokine ligand
CXCL: C-X-C motif chemokine ligand
DED: dry eye disease
ERK: extracellular regulated protein kinase
ICAM-1: intercellular adhesion molecule 1
IFN-γ: interferon-γ
IL: interleukin
JNK: jun N-terminal kinase
LFA-1: lymphocyte function associated antigen 1
LFU: lacrimal functional unit
MAPK: mitogen-activated protein kinase
MGD: meibomian gland dysfunction
MHCII: major histocompatibility complex class II
MMP: matrix metalloproteinase
NF-κB: nuclear transcription factor-κB
NK cells: natural killer cells
NLRP3: NLR family pyrin domain containing 3
NSAIDs: non-steroidal anti-inflammatory drugs
PRR: pattern recognition receptor
TCM: Traditional Chinese Medicine
TGF: transforming growth factor
Th: T helper
TLR: Toll-like receptor
TNF-α: tumor necrosis factor-α
Tβ4: thymosin β4.
